# A Brief Review on Biomimetics 3D Printing Design

**DOI:** 10.3390/biomimetics10100647

**Published:** 2025-09-26

**Authors:** Rúben Couto, Pedro R. Resende, Ricardo Pinto, Ramin Rahmani, João C. C. Abrantes, Iria Feijoo

**Affiliations:** 1 Grupo E-Materiais, Departamento de Ingeniería de Materiales, Mecánica Aplicada y Construcción, EEI, Universidade de Vigo, 36310 Vigo, Spain; 2 proMetheus, Escola Superior de Tecnologia e Gestão, Instituto Politécnico de Viana do Castelo, 4900-347 Viana do Castelo, Portugalramin.rahmani@citin.pt (R.R.);; 3 CEFT, Department of Mechanical Engineering, University of Porto, 4200-465 Porto, Portugal; 4 CiTin—Centro de Interface Tecnológico Industrial, 4970-786 Arcos de Valdevez, Portugal

**Keywords:** biomimetic, additive manufacturing, 3D printing, selective laser melting

## Abstract

Over millions of years of evolution, nature provided tools to optimize different functions in animals and plants. Different strategies observed in nature serve as models for solving complex engineering problems. Additive manufacturing (AM), also known as 3D printing, enables us to produce shapes that would not be possible with traditional subtractive manufacturing. In this way, it is possible to produce complex detailed shapes using an automatic process. Biomimetics involves drawing inspiration from nature and applying it to solve specific engineering challenges, often with the goal of optimization and enhanced performance. Three-dimensional printing enables the replication of complex natural shapes, opening new avenues for innovation. In this paper, we review the state of the art in biomimetics, including studies on mechanical properties, design strategies, manufacturing techniques, and the use of composites.

## 1. Introduction

Animals and plants have been developing their capabilities to survive and prosper in the environment through natural selection. As humans, mimicking nature to build and fulfill our needs is not a new concept. We have been inspired by nature on both macro- and nanoscales, Table 1. Some simple examples include the pyramids, which were probably based on the shape of mountains. Umbrellas were invented by Lu Ban around 1700 years ago while observing children taking shelter in lotus leaves [1]. In the 1950s, Otto Schmitt made the first reference to the word “Biomimetics”.

Synonyms of this term include “biomimesis, biomimicry, bionics, and biologically inspired design”. Leonardo da Vinci was inspired by birds to design some machines for flight [2]. The Wright brothers mimicked the method where birds use air currents to gain lift force and produce directional changes. Velcro is another product with biological inspiration, which was created after George de Mestral noticed burs stuck on his clothes and the fur of his dog. He realized that the bur’s surface had several tiny hooks [1].

Biomimetics might have started with basic stone shelters such as caves, which evolved to individuals building temples inside of them. Another example is silk, one of the first materials made by humans, which was inspired by silk worms. A more well-known milestone is the pyramids, whose shapes might have been inspired by the shape of mountains. The umbrella was invented after an individual in China observed children protecting themselves from the rain with lotus leaves. Around the 1500s, Leonardo da Vinci designed a flying machine inspired by birds [1].

In the post-Industrial Revolution period, several significant examples can be identified, Table 2, one of which is the Crystal Palace.

The Wright brothers were inspired by how birds use air currents to gain lift. Velcro was invented after observing burs being attached on a dog’s fur. The concept of a Circular Economy refers to planning businesses without waste, as in nature, there is no waste.

The study of patterns in nature has been noted in examples such as the “golden ratio” and Fibonacci numbers [3,4,5]. The golden ratio has been studied since around 450 BC, and Fibonacci numbers have been of interest since about 1200 AD [5,6,7,8].

Another example of biomimetics is in its application to reduce drag, whereby small indentations are created on a surface, similarly to the small “hooks” on a shark’s skin. These small bumps are known as “riblets”.

Observing how a school of fish swam close together helped researchers to make wind turbines operate very close each other. This involved making a vertical design to reduce the chaotic movements of the traditional designs, which also increased the efficiency of the system.

Geckos can climb because of small hair-type structures known as setae, which inspired the creation of an adhesive substance that does not rely on typical viscoelasticity, but on a concept known as draping adhesion [9,10,11,12].

There is a city in India that has been planned based on biomimetics, where there are green areas and systems to collect rainwater. The Nanoscale Surface Textures are created by the company Fusion Bionics, who were inspired by textures from nature to apply, for example, anti-icing textures for aviation, as well as antibacterial properties for medical products, which were based on the denticles of shark skin. The textures are created with Direct Laser Interference Patterning (DLIP) [1,13].

**Figure 1:**
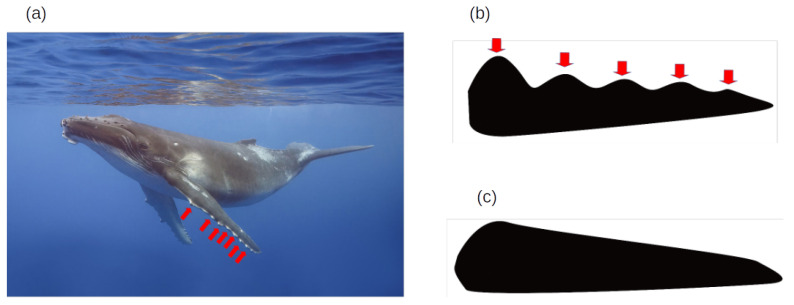
Humpback whales (*Megaptera novaeangliae*) are characterized by the presence of tubercles, rounded bumps, along the leading edges of their pectoral fins (flippers) (**a**). The image is adapted from Charles J. Sharp (2024) and licensed under the Creative Commons Attribution-Share Alike 4.0 International [15]. The red arrows were added to the original image to indicate the tubercles. An illustration by Rúben Couto shows how this feature has been adapted to the design of wind turbine blades, a concept pioneered by WhalePower Corporation (**b**). In contrast, blades with conventional smooth edges are shown for comparison (**c**).

A more complex example is the biomimicry based on humpback whales (*Megaptera novaeangliae*), where their fins were used as the inspiration for wing-like structures that can be applied to wind turbines, as is exemplified in Figure 1; in this way, they can obtain energy with efficiency [14].

**Figure 2:**
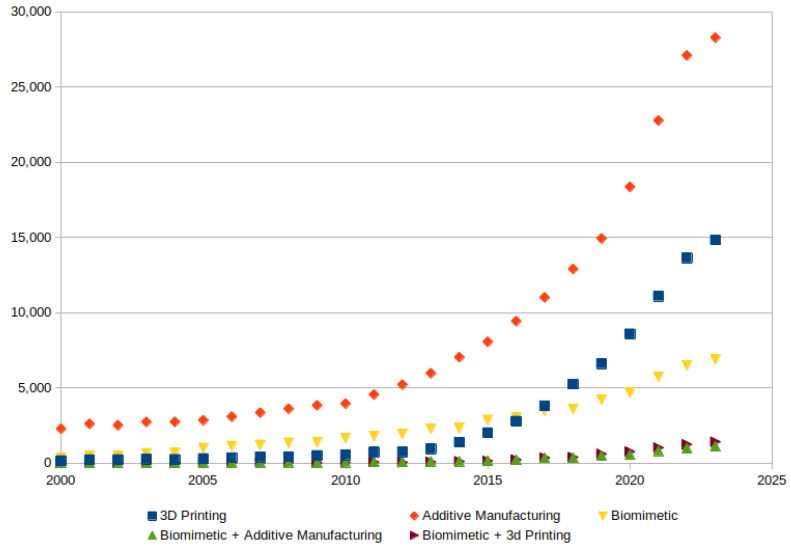
Evolution of published papers by keywords such as “Additive Manufacturing”, “Biomimetic”, “3D Printing”, and a combination of words. Graphic generated by the authors.

Since 2000, the interest in additive manufacturing, 3D printing, and biomimetics has increased exponentially, showing that these fields of study have been promising in academic research [16]. The literature was searched using keywords on ScienceDirect, an online platform for scientific and technical articles, https://www.sciencedirect.com/, as shown in Figure 2. With this interest, more attention has been given to materials and shapes based on nature [17,18,19].

## 2. Materials for Biomedical and Biomimetic Applications

The selection of suitable materials is critical in biomedical and biomimetic additive manufacturing, as it directly influences biocompatibility, mechanical performance, and functional integration with biological systems. Current research explores a broad range of metals, polymers, and composites, both established and emerging, to meet the diverse demands of applications such as implants, tissue scaffolds, and bio-inspired devices. This section provides an overview of the main classes of materials used today and those under investigation for next-generation biomedical solutions.

### 2.1. Metals

Titanium and Ti-6Al-4V alloys are widely used for load-bearing implants because they offer a high strength-to-weight ratio, outstanding corrosion resistance, and excellent biocompatibility. Additionally, porous structures created through powder bed fusion (PBF) improve bone integration and help minimize stress shielding [20].

Zinc and its alloys have gained significant attention in the field of additive manufacturing for medical applications. This interest stems from zinc’s natural biodegradability, favorable mechanical properties, and excellent biocompatibility. Additive manufacturing of biodegradable zinc-based metals provides key benefits for producing customized medical implants tailored to individual patient needs [21].

### 2.2. Polymers

Common biopolymer materials (PLA, PCL, PLGA, and PEG) are widely used for tissue engineering scaffolds, drug delivery systems, and biodegradable implants. They offer tunable degradation, high printability, and customizable porosity [22]. Shape-memory polymers (SMPs) are in the emerging field of 4D printing, enabling minimally invasive implantation, transforming in response to body temperature, promising for stents and tailored biomedical devices [23].

### 2.3. Composite Materials

Metal Matrix Composites (MMCs) combine metals like Ti-6Al-4V or stainless steel with bioactive ceramics (e.g., hydroxyapatite, TiC, or SiC). These improve mechanical properties and promote bone ingrowth, with studies showing enhanced wear resistance and mechanical performance [24].

Bioceramic composites, such as HA–polymer, BG–polymer, or bioactive glass-reinforced scaffolds, support tissue integration and match the chemical properties of bones [25].

Polymer–metal composites, which embed metal or ceramic particles into polymer matrices, are gaining traction for improved bioactivity, mechanical strength, and antimicrobial function [26].

## 3. Additive Manufacturing Techniques and Printed Model Geometries

Table 3 presents examples of previous studies employing various 3D printing technologies and materials.

Many works used metal 3D printing based on biomimetics, such as  the honeycombs, as shown in the table below, or when studying the xylem of plants; this is carried out so that high power densities and open-circuit voltages can be manufactured. After studying the lungs, similar mechanisms were applied for uniform reactant distribution across the electrodes.

**Table 3:** Prototypes, 3D printing techniques, materials, properties, and applications studied.

Biomimetic Prototype	3D Printing Technique	Materials	Properties	Applications	Refs
Xylem with aligned channels	SLM	Metal powder	High power density, open-circuit voltage	Zinc–air battery	[27,28]
Lung	DMLS	Stainless steel powder	Uniform reactant distribution across the electrodes	Fuel cell	[29]
Honeycombs	FFF	PLA	Stiffness	Energy absorption/biomedical applications	[30]
Voronoi Tessellations	SLM	Grade II titanium	Lightweight macrostructures	Cranial prostheses	[31]
Mandibular Models	SLS	PA 2200	RMS values from STL data and 3D-printed models	Accuracy/trueness	[32]

For example, regarding the Voronoi Tessellations [31], mechanical testing has not been conducted, but it would be interesting to carry out, for example, fatigue or compression tests, to optimize some complex shapes that are meant to be used in human body with the use of prosthesis. The lack of mechanical tests in prosthesis might be an opportunity for further research. In this paper, there is an interesting experiment where the minimum thickness requirement used is 0.4 mm.

RMS values were analyzed using STL data and 3D-printed models [32], and the best accuracy was with SLS printers with the highest overall trueness (RMS 0.11 ± 0.016 mm), as well as the FFF 3D printer with the highest overall precision (RMS 0.05 ± 0.005 mm). Once again, no mechanical testing has been conducted in human prostheses, nor have there been optimization experiments to have a lighter weight or similar density to human bone, or even proper mechanical properties.

### 3.1. Mechanical Testing and Its Relevance to Human Prosthetics

#### 3.1.1. Compression Testing

Compression testing is crucial for simulating weight-bearing loads in bones and implants. Cortical bone exhibits a compressive strength of ~133–193 MPa, making compression testing essential for validating load-bearing prosthetic components.

#### 3.1.2. Tensile Testing

Tensile testing is important to assess how materials respond to stretching forces. Human cortical bone has a tensile strength ranging from ~51 to 133 MPa. This test reflects how prosthetics may perform under limb movement and stress conditions [33].

A Zn-Mg-Mn alloy exhibiting a tensile strength of 414 MPa and an elongation of 26% was developed, with better mechanical properties than other Zn-Mg-Mn alloys. These findings demonstrate that Zn alloys hold great potential as orthopedic implants due to their excellent mechanical integrity [34].

Cortical bone typically exhibits an elastic modulus in the range of 10–30 GPa, whereas conventional metallic implant materials like titanium (~110 GPa) and cobalt–chromium alloys (200–230 GPa) are considerably stiffer. This mismatch in stiffness can hinder proper load transfer to the surrounding bone, increasing the likelihood of stress shielding and associated bone resorption [35].

The quasi-static Young’s modulus and yield strength of porous Ti6Al4V alloys with relative densities between 30% and 70% range from 6 to 40 GPa and from 100 to 500 MPa, respectively. Their quasi-static compressive properties can be precisely adjusted by controlling porosity to closely mimic those of cortical bone. Additionally, the strain rate sensitivity of these porous alloys is influenced by their porosity [36].

#### 3.1.3. Shear Testing

Trabecular bone shear strength was assessed using micro-CT-based models of 54 specimens (5 mm cubes). For cortical bone, shear strength (longitudinal) is 51.6 MPa [37], and shear strength (anisotropy) is ~50 MPa [38]. The average shear modulus ranged from 5.44±0.64GPa to 6.97±0.70GPa, while the average torsion strength varied between 47.92±7.80MPa and 66.31±7.51MPa [39].

#### 3.1.4. Limitations of AM Materials

While AM metals show sufficient static strength, fatigue resistance (under cyclic loading) is often lower than conventionally manufactured components due to surface defects or anisotropy. Biodegradable implants degrade over time, potentially compromising mechanical integrity before the tissue has fully healed.

According to Table 1, Table 2 and Table 3, the integration of biomimetics with 3D printing design, as discussed in [40], must be a convergence of sustainability, human-centric technologies, and digital transformation [41]. By mimicking nature’s time-tested designs through AM, engineers can create more efficient, adaptive, and resource-conscious solutions, significantly reducing material waste and energy consumption, key pillars of sustainability [42]. This approach also advances human-centered technologies by enabling the design of customized, high-performance products tailored to individual needs, such as prosthetics, wearable devices, and bio-inspired systems that improve quality of life.Furthermore, the reliance on digital modeling and automated production in 3D printing exemplifies the core of digital transformation, where intelligent design and data-driven fabrication processes are revolutionizing how we innovate across industries [43]. The output of such industry evolution is inspired by metallic lattices like triply periodic minimal surfaces (TPMSs), which are attractive due to their lightweight scaffolds, biomedical applications, and complex shapes manufactured by AM [44].

### 3.2. Biocompatibility of 3D-Printed Metallic Specimens

This section addresses the biocompatibility of metallic components produced by additive manufacturing (AM), with a focus on wear resistance (bio-tribology), cytotoxicity, surface roughness, and other critical performance metrics that extend beyond mechanical characterization.

#### 3.2.1. Wear Resistance and Tribocorrosion

Wear resistance is essential in 3D-printed metallic implants, particularly for orthopedic and dental applications subjected to cyclic loading and corrosive environments. Wear can generate metallic debris that triggers inflammation or osteolysis, potentially compromising implant integration and long-term performance. Surface hardening treatments and biocompatible coatings, such as diamond-like carbon (DLC), titanium nitride (TiN), and ceramic layers, have been shown to mitigate tribocorrosion and extend implant service life [45]. In Table 4, a comparison of selected properties of biomaterials shows their strength–weight ratios (MPa/g/cm^3^), Elastic Moduli (GPa), and their main key applications as biomaterials.

#### 3.2.2. Cytotoxicity and Ion Release

While titanium-based alloys such as Ti-6Al-4V are widely accepted in biomedical use, other shape memory alloys like NiTi (Nitinol) produced by LPBF are gaining attention for their unique biomechanical properties. However, NiTi raises biocompatibility concerns due to nickel ion release, which can induce cytotoxicity or allergic responses. A study by [46] evaluated in situ alloyed NiTi fabricated via LPBF and showed promising results. Although LPBF-fabricated components made from elementally blended pure Ni and Ti exhibit slightly lower corrosion resistance compared to those produced from pre-alloyed NiTi powders, both materials demonstrate comparable biocompatibility, particularly with respect to cytotoxicity. The study highlights that using elementally blended pure nickel and titanium powders in LPBF yields promising biocompatibility and corrosion resistance. Their low cytotoxicity and strong passivation behavior suggest potential for biomedical use. Further research is recommended to optimize LPBF processing and post-processing steps, aiming to enhance microstructure-dependent corrosion resistance while reducing manufacturing costs [46].

Numerous in vivo and in vitro studies have confirmed that additively manufactured (AM) components exhibit biocompatibility comparable to those produced by conventional manufacturing methods. The effects of both the raw materials and the fabricated Ti6Al4V bulk structures on NIH 3T3 (fibroblast cell line derived from embryonic mouse) were examined. After 24 h of incubation, no cytotoxic effects were detected, and all tested Ti6Al4V samples maintained cell viability above 80%, indicating good biocompatibility of the AM-processed material [47].

#### 3.2.3. Surface Roughness and Topography

Surface topography directly influences cell adhesion, proliferation, and osseointegration. Both average roughness (Ra) and peak–valley morphology affect cellular responses and bone tissue ingrowth. These features depend on the AM process (e.g., SLM and EBM), post-processing methods (e.g., polishing and etching), and chemical composition. The current literature lacks consensus regarding surfaces with roughness values greater than 5 μm. Additive manufacturing techniques often produce surfaces with average roughness around 25 μm, which closely resemble the architecture of trabecular bone. This similarity suggests a potential to enhance new bone formation due to improved biomimicry. Surface polishing reduces the risk of fatigue-related failures by eliminating stress-inducing factors found in uneven regions. It can be combined with physical, chemical, or biological treatments to enhance surface quality and performance [48]. For example, smoother surfaces (Ra∼0.05 µm) are preferred for low-friction interfaces (e.g., femoral heads) [49]. A porosity level between 40% and 60% in 3D-printed porous titanium samples was identified as optimal for biomedical applications [50]. In high-purity magnesium scaffolds, a surface roughness (Sa) of 1–2 is deemed suitable for clinical use and aligned with medical standards [51].

#### 3.2.4. Additional Bioperformance Metrics

Further parameters impacting biocompatibility might include the following:

Electrochemical corrosion in simulated body fluids is often measured via electrochemical impedance spectroscopy (EIS) or polarization tests. Higher porosity and surface roughness tend to increase corrosion rates [50].

The study tested 3D-printed titanium implants with a silver coating to determine whether they are safe and suitable for biomedical use. Researchers grew bone-forming cells (osteoblasts) and human skin cells on the implants for 14 days and found that the silver coating did not harm the cells. In fact, the surface supported good cell growth and showed signs of promoting bone-related activity. The results suggest that these implants are both biocompatible and may help reduce infection risk, making them promising for future medical applications [52].

Bioactive and antimicrobial coatings (e.g., silver nanoparticles and polydopamine–hydroxyapatite composites) can prevent infections while supporting cellular viability, provided that their concentration and release kinetics are carefully optimized to avoid cytotoxic effects [53].

## 4. Sample Geometries: Tension, Compression, and Shear Testing in Biomimetic 3D-Printed Metals

### 4.1. Tensile Testing

Tensile testing of metallic materials is usually performed using dog-bone-shaped specimens according to standards such as ASTM E8/E8M-24—Standard Test Methods for Tension Testing of Metallic Materials or ISO 6892-1—Metallic materials—Tensile testing—Part 1: Method of test at room temperature [54], which define the geometry and procedures for uniaxial tension tests. These tests examine fundamental mechanical properties such as elastic modulus, yield strength, tensile strength, elongation at break, and reduction in the area. However, tensile testing alone does not represent the full range of physiological stresses experienced by implants or prostheses. Studies using 3D printing were carried out with classical dog-bone-shaped specimens, which can inspire new experiments with SLM metal; for example, different materials (ABS and Resin) were compared and the infill of the inside volume of the specimens was varied [55]. Furthermore, material properties and microsctrutural changes were investigated on FFF 3D printers, for PLA and Tough-PLA [56]. Microstructural changes, rupture studies with SEM, and different fillings can be studied with the SLM 3D printer. Also, there is an FFF 3D printer for metal, instead of SLM, where 3D-printed angles of the specimens were varied, and then the mechanical properties were studied using tensile tests [57].

### 4.2. Compression Testing

Compression testing is especially important for porous biomimetic structures and is standardized, for example, by ASTM E9—Compression Testing of Metallic Materials (room temperature) [58] for metallic materials. Typical specimens are cylindrical or cubic, and the test evaluates compressive modulus, yield strength, and failure mechanisms. In additively manufactured lattices (e.g., titanium alloys such as Ti-6Al-4V), compression testing is critical, as these structures are often used in load-bearing implants (e.g., femoral stems and vertebral cages) where compressive loads dominate. Test dimensions such as 10 mm^3^ lattice cubes are commonly used to avoid buckling and ensure uniform stress distribution.

### 4.3. Shear Testing

Shear testing captures the material’s resistance to sliding forces, which are particularly relevant at implant–bone interfaces or in modular prosthetic joints. There are available methodologies, such as ASTM D5379—Shear Properties of Composite Materials by the V-Notched Beam Method [59] and ASTM D3410—Shear Loading Compression [60] (combined loading compression), that provide useful references. For metallic 3D-printed geometric samples, examples can be found in literature, including pure shear flat plate specimens [61,62]; V-notched samples were also used for shear tests [63].

In Table 5, a summary is provided of the mechanical test types, applicable standards, specimen geometries, and reference features associated with 3D-printed metal biomimetic structures.

**Table 5:** Mechanical test types, standards, specimen geometries, and reference resources relevant to 3D-printed metallic biomimetic structures.

Test Type	Standard	Specimen Geometry	References Link	Application/Notes
Tension	ASTM E8/E8M, ISO 6892-1	Dog bone (flat or round)	[64,65,66]	Measures tensile strength, elastic modulus, ductility
Compression	ASTM E9	Cylindrical or cubic (e.g., $10{\mathrm{mm}}^{3}$ lattice)	[64,67]	Used for lattice structures under load; avoids buckling
Shear	ASTM D5379, ASTM D3410 (composites)	V-notched beam or pure shear flat plate	[61,62,63]	For implant–bone interface or modular component stress; adapted for metals
Lattice mechanics study	Non-existent standard	10 mm^3^ lattice cubes (compression) and pin-loaded tensile specimens	[67]	Application to Ti-6Al-4V biomimetic implants manufactured by LPBF

## 5. Three-Dimensionally Printed Biomimetic for Biomedical Application

There are various types of biomedic applications using biomimetics, including implants, lab-on-chip, and artificial organs or tissues. The complex structure of the human body is a challenge that requires specific criteria for medical devices, where traditional manufacturing is unable to succeed. For example, there is the need to have 3D porous structures with proper mechanical properties to allow tissues to regenerate. Holes with the correct sizes allow researchers to create an environment for cells to grow [68].

There are artificial lung networks that have been printed by SLA technology [69], as well as Limpet Tooth-Inspired microneedles printed by the MF-3DP process for drug delivery [70], bioprinted corneas with a collagen-based bio-ink [71], and a 3D-printed biomimetic mussel-inspired scaffold for tissue regeneration [72].

Some approaches are looking into biodegradable metal materials, such as Mg, Zn and Fe, because implants with Ti alloys, stainless steel, and CoCrMo might produce stress-shielding effects in some circumstances and generate toxic metal ions, which might lead to removal of the implant. The incorporation of nanomaterials into 3D-printed polymers has been studied to develop composites suitable for biomedical applications. There is a study on 3D-printed implants, where the angle of manufacturing is taken into account, which affects the mechanical properties, and prior to this method, a finite element analysis (FEA) was carried out. In the same study, lattice patterns were used to emulate natural structures to augment biocompatibility, osteogenic performance, and mechanical properties. In addition, the different printing angles were studied [73].

There is also a study where the best specimens were printed with a 50° angle [73].

Another article [74] also printed different specimens at different angles and with different wall thicknesses.

Again, printing angle was taken in account like in this study for modified honeycomb parts of Ni-15Fe-5Mo, as well as printing different specimens with different wall thicknesses. In this study, 30° was the angle where better mechanical properties were seen, with high strength and higher elongation, no matter the wall thickness.

The reason for this is due to the fact that the weakened molten parts are not parallel or perpendicular to the stress direction action. But the relation between the orientation of the polycrystalline grains and stress direction was more problematic for thin walls with 30° angles.

This study was with carried out with the intention for applications in magnetic shields. The appropriate laser heat source setting was studied, as it has been noted as an important part of this subject. Because there are gaps between the powder particles, the laser beam is reflected in different directions, which means that there is less energy distribution at the bottom of the powder layer.

## 6. Heat Treatment

The products from LPBF show defects, such as lack of fusion, residual thermal stress, and anisotropy. Some post-processing techniques, such as electrochemical etching and a femtosecond laser, were applied to create micro- and nanosurfaces to improve the ability of water to spread on the surface [75]. Heat treatment on 3D-printed LPBF-spread specimens were conducted to enhance the mechanical properties [76], where the strength was significantly increased and the anisotropy was eliminated in the strength levels.

The authors realized that building orientation did not significantly affect the microstructure, hardness, or notch toughness. Another study with fatigue SLM 3D-printed specimens post-treatment shows that the most important factors of the fatigue behavior of maraging steel are keyhole pores, a lack of fusion voids, surface finish, and microstructural and residual stresses [77].

For maraging steel, higher strengths and hardnesses were reached compared to conventional steel without thermal treatment and with treatment for 4 h at 500 °C [78].

A study [79] on laser surface treatment (Biomimetic Laser Surface Treatment (BLST)) noted that changing the parameters of the laser will alter the surface morphology of specimens, as well improve the wear of the specimens. They also indicate that tiny biomimetic scales can protect the specimens. These methods were based on pangolin scales and were used to prepare a surface of specimens with a large amount of manganese steel through a pulsed radiation mode CO_2_ laser. Here, a hardening furnace is not used, but laser is employed to enhance the mechanical properties. This technique shows high flexibility and adaptability, allowing us to heat certain parts with precision.

In another study [80], modifying the titanium surface by anodic oxidation and alkali heat treatment is mentioned, which generates a nanonet topography and a hydrophilic surface. This study focused on the surface modification for implants printed in titanium with SLM, with the goal of bone regeneration.

Another study applied heat treatment on LPBF porous titanium implants, registering an increase in the elastic modulus, and annealing at 675 °C for 1 h, which reduced the deformation of bone implants [81].

Post-processing of 3D-printed metals often includes heat treatments tailored to the material’s microstructure and intended mechanical performance. In addition to ferrous alloys, non-ferrous systems such as cobalt–chromium (Co–Cr) and nickel–titanium (Ni–Ti) also benefit significantly from thermal treatments. For example, solution treatment followed by aging can refine the microstructure of Co–Cr and reduce internal stresses, improving fatigue resistance and corrosion behavior [82]. In one study, the in situ heat treatment approach was integrated during the directed energy deposition (DED) of NiTi shape memory alloys, which refined the microstructure, enhanced phase transformation characteristics, and reduced the need for conventional post-process annealing [83]. Wang et al. (2025) investigated the effects of solution and aging heat treatments on the microstructure and residual stresses of a Ni-Co-based superalloy fabricated via laser powder bed fusion (LPBF). The heat treatments promoted recrystallization, grain refinement, and γ’-phase precipitation, effectively reducing the residual stress levels [84]. These steps are critical to tailor functional properties for biomedical applications such as stents or orthodontic wires.

Moreover, for polymer-based additive manufacturing, annealing protocols are often applied to reduce residual stresses, enhance crystallinity, and improve dimensional stability. For PLA, annealing was performed at temperatures ranging from 70 °C to 110 °C, with treatment times between 40 and 200 min. The optimal conditions were identified at 90 °C for 120 min, where the most significant effects were observed [85].

One study evaluated the effects of annealing on PETG and its composites reinforced with carbon and Kevlar fibers. Annealing improved mechanical performance across all materials, notably increasing hardness, bending strength, and the modulus, while also reducing stress relaxation and creep, with the most significant gains observed in the fiber-reinforced samples. The dimensional tolerances were changed after the process [86]. This process improves strength and increases thermal resistance, particularly in high-performance polymers used in aerospace and medical devices.

## 7. Honeycombs

**Figure 3:**
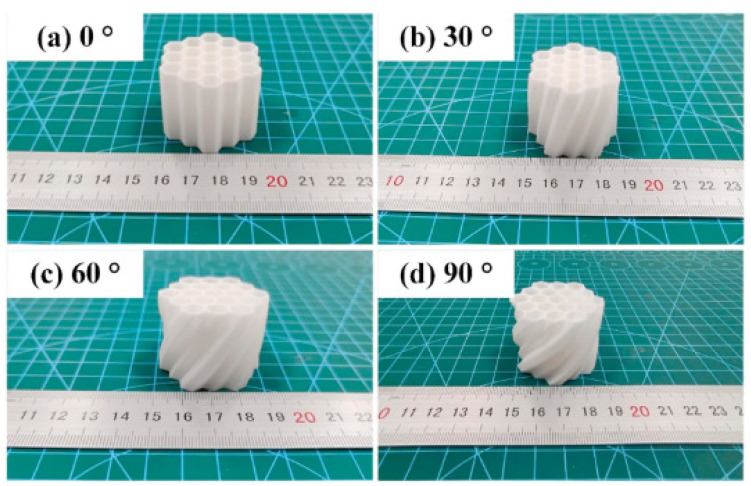
Twisted honeycombs (THCs) with different angles, for compression tests, yielding the best SEA performance at 30°. Reproduced with permission from Anfu Guo [89].

Honeycombs are natural macroscopic structures that resemble other natural but microscopic structures as iris leaves, cork, or even balsa wood [87]. Wood and bones have lattice structures and mechanical behaviors such as being natural energy absorbers. The optimal design of cellular structures is fundamental to influencing the load transfer and reducing damage severity [88]. The hexagon structures has lightweight characteristics, good stiffness, great strength, and high specific energy absorption (SEA) [89]. Studies of mechanical properties have been carried out for structures based on 3D-printed honeycombs, where elastic properties such as the elastic modulus, Poisson’s ratio, and yield stress were analyzed in their two major in-plane directions. As shown in Figure 3 [89], the authors investigated twisted honeycombs to study specific energy absorption (SEA). The structures were twisted at different angles, with the best SEA observed at 30°. This work addresses the lack of studies on the influence of the twisted angle along the Z-axis. Interestingly, the value of 30° appears again, similar to the previously mentioned study [74], which was conducted using T-bone-shaped specimens under tensile testing.

**Figure 4:**
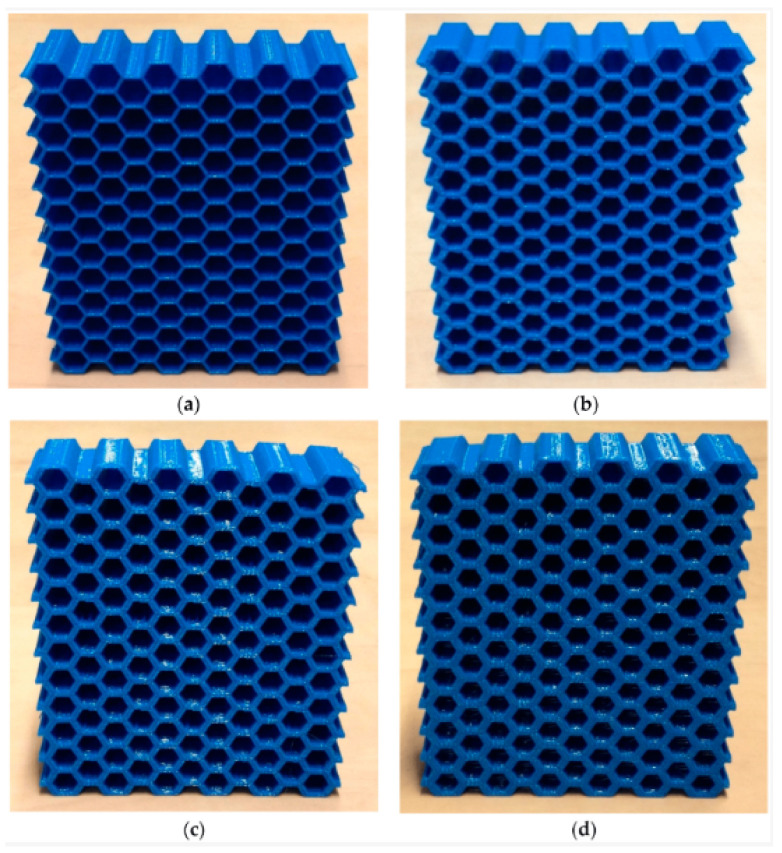
Three-dimensionally printed honeycomb samples made of PLA, showing the variation in the thickness-to-length ratio (t/l) of the cell walls, where t is the wall thickness and l is the cell length. (**a**) t/l = 0.09, (**b**) t/l = 0.18, (**c**) t/l = 0.27, and (**d**) t/l = 0.36. Reproduced from [30]. Distributed under the Creative Commons Attribution (CC BY 4.0) license.

In another study [30], as shown in Figure 4, the stiffness matrices were studied with Euler–Bernoulli (not applicable to thick honeycombs) and Timoshenko beam theories. The results were very close to the laboratory tests. In this specific study, they established a relation between the Elastic Modulus and density.

### 7.1. Tensile

According to the published study [90], specimens were printed for tensile testing. Honeycombs were incorporated in the gauge section of the specimens made of titanium alloy Ti6Al4V and stainless steel 316L. Stress–strain curves were analyzed, along with micrographs of the fracture surfaces for both Ti6Al4V and 316L specimens. There is also the possibility to study these types of specimens under fatigue testing, as well to examine their fracture micrographs.

### 7.2. Compression

**Figure 5:**
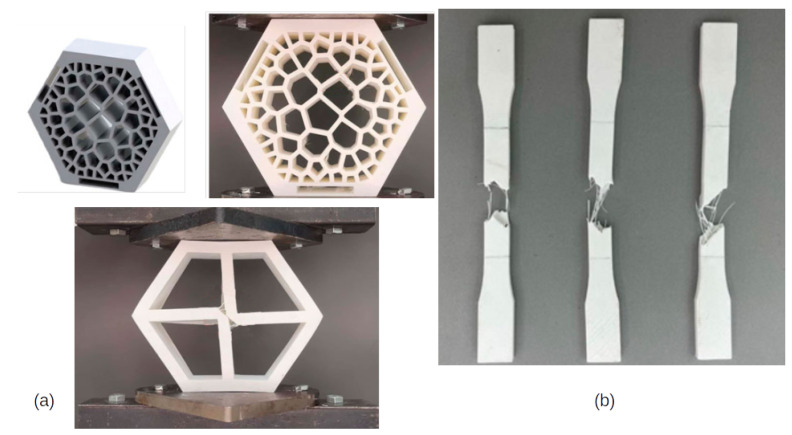
Specimens used in the study [91]. Compression tests were performed with the honeycombs (**a**), and tensile tests were performed with printed Polylactic Acid (PLA) tensile specimens (**b**). Adapted from [91]. Used under Creative Commons CC BY license.

In another study [91], honeycombs were created, but the goal of the study was to fill them with Voronoi Tessellations. Compression and tensile tests were carried out to examine the properties of Polylactic Acid (PLA), yielding a range of data, including elastic modulus of 1003.95 ± 11.09 MPa and ultimate tensile strength of 41.54 ± 2.02 MPa, respectively, as shown in Figure 5.

A study [92] on  single metallic and polymeric honeycombs, with four different 3D-printed hexagonal honeycomb geometries, was conducted, where compression tests were used to make comparisons between FDM and DMLS (Direct Metal Laser Sintering) over these four different honeycomb configurations.

It is possible to repeat this study with the Voronoi Tesselations or fractal filling inside one single honeycomb, which is similar to this approach, but with a metallic 3D printer, as well to study the elasticity modulus of tool steel or other powders, for compression testing or with tensile specimens.

**Figure 6:**
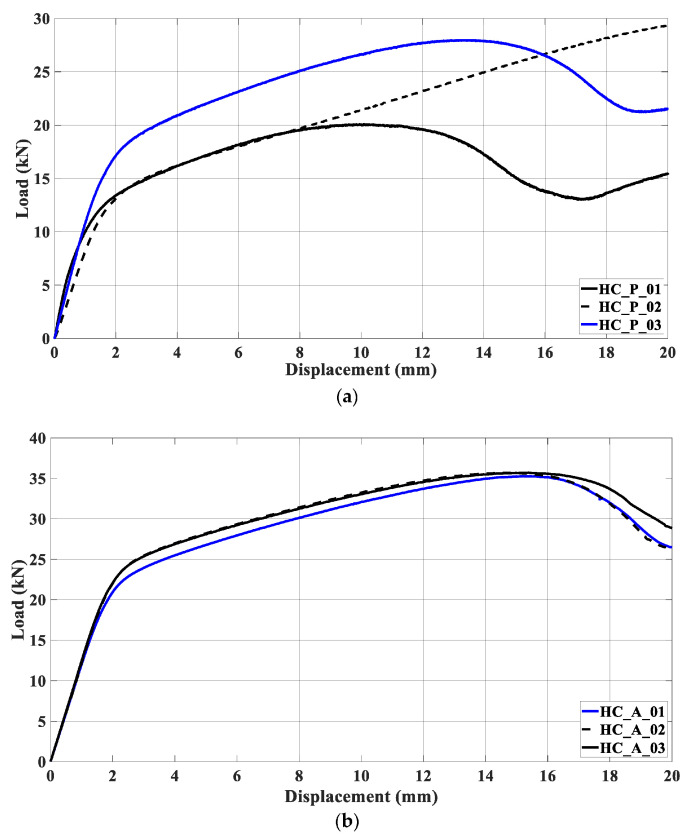
Compression tests used in the study [92], with 3D-printed filling of honeycombs of PETG (**a**) and ABS (**b**), without a metallic external layer. Adapted with permission from Rita de Cássia Silva [92].

In another study, specimens with a group of honeycombs were built in PETG and ABS, which was combined with a metallic external part. Compression tests and tensile tests were conducted with different specimens to determine “flow stress, tensile strength at break, modulus of elasticity, elongation, ultimate strength, deflection stress, and compressive modulus” [92]. Figure 6 shows how the material responded under compression by illustrating the relationship between the applied load and the resulting displacement.

**Figure 7:**
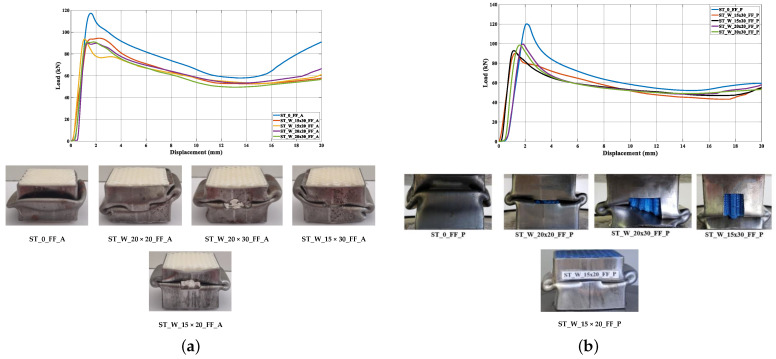
Compression tests with five different metallic shells filled with honeycombs made of PETG (**b**) and ABS (**a**) used in the study [92]. Adapted with permission from Rita de Cássia Silva [92].

Therefore, tests were conducted with a metallic layer, Figure 7. The different results can be observed depending on the type of filling for both filaments. The naming follows the format ST_W_DD_FF_MM, where ST stands for the steel tube, W indicates the window and its dimensions (e.g., W_15 × $20{\mathrm{mm}}^{2}$), FF is the filament type (PETG or ABS), and MM refers to the material specification, including the type of honeycomb and filament. The behavior was identical in the elastic phase. The ABS specimens showed a more uniform response in the elastic zone compared to PLA.

A similar study can be conducted with tool steel specimens. They can be printed with 100 % metallic infill honeycombs.

#### MetallicPrinting of Honeycombs

Some tests with honeycombs were conducted with tool steel with laser powder bed fusion (L-PBF), which used simplified 2D finite element (FE) analysis to determine the stress distribution. Compression tests were performed [93].

The previous paper served as the basis for another study [94] that employed the digital image correlation technique and provided a more detailed analysis of the honeycombs.

**Figure 8:**
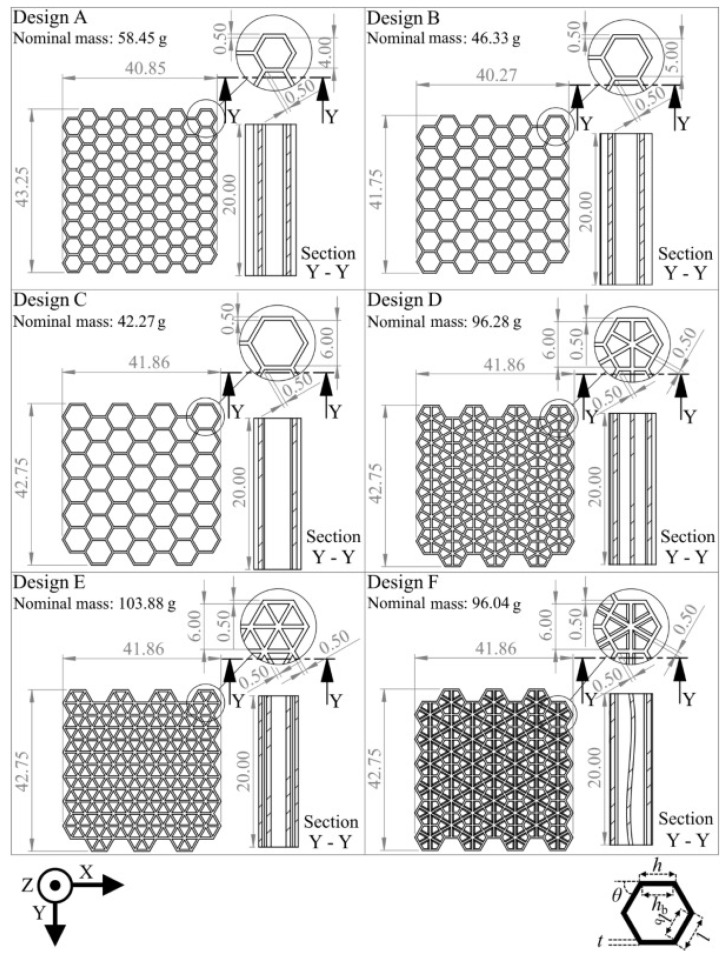
Different honeycomb designs for compression tests, labeled from A to F, with wall thickness of t=0.5mm. Reproduced from [93]. Distributed under the Creative Commons Attribution (CC BY 4.0) license.

**Figure 9:**
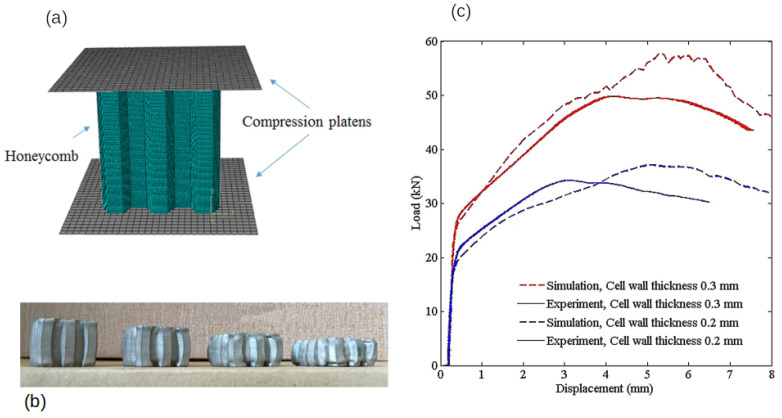
Results from the study [95]: (**a**) numerical model for compression of honeycomb specimen; (**b**) the specimens underwent compression testing; and (**c**) comparison of experimental vs. simulation values for wall thicknesses of 0.3 mm and 0.2 mm. Adapted with permission from K. Chandrashekhara [95].

In Figure 8, each design varies in cell height, outer width (*h*), and internal width ($h_{b}$), as well as structural configuration, resulting in different nominal masses. The specimens are shown from the front and in section Y–Y, along with the height and width values for each design Compression in another plane was also conducted to study how honeycombs fractured, with the conclusion that the failure was initiated by the plastic failure in the cell walls. Specimens were printed by selective laser melting (SLM). In Figure 9, comparisons with predicted and tested values were made.

A non-linear model was made to describe the behavior of the material in the plastic zone. The damage progression was influenced by the direction of the printing [95].

## 8. Plant Stem Profiles

A study compared the profiles with different geometries for a study on the efficiency of materials with the plant motherwort (Leonurus cardiaca) [96], where the stems are divided between hollow internodes and solid nodes, making it a lightweight model. Different profiles were produced with mechanical testing. Torsional tests were conducted and organized into profiles, providing a different perspective from the usual tensile, bending, or compression testing in biomimetic specimens.

**Figure 10:**
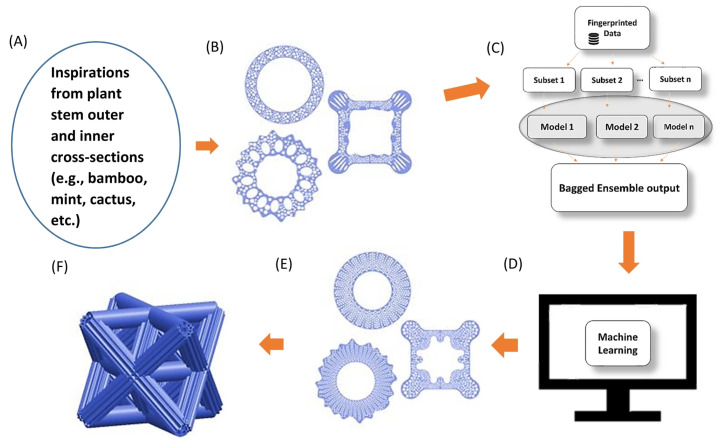
Machine learning framework based on plant stems. (**A**) Nature structure inspirations, (**B**) first designs, (**C**) machine learning trees, (**D**) data filtering, (**E**) CAD design of optimized rod, and (**F**) potential application (structure with biomimetic rods). Reproduced with permission from Guoqiang Li [97].

In [97], the authors manufactured columns or rods with better buckling resistance based on plant profile stems. Machine learning was applied after different digitalized biological structures were determined for each feature. The database was built to determine a relation between the structural features and buckling load. A filter was applied to the data for optimization. After this process, FEA (finite element analysis) was applied to obtain buckling loads. Euler buckling equations could not be used in this case because the complex geometrical shapes were difficult. This was a reason why they used FEA to feed the training database. Machine learning made optimizing and finding biomimetic rods faster. Rods were 3D-printed, tested, and then compared to the modeled results. The FEA was validated and used to calculate the buckling load of optimized biomimetic rods, as exemplified in Figure 10.

**Figure 11:**
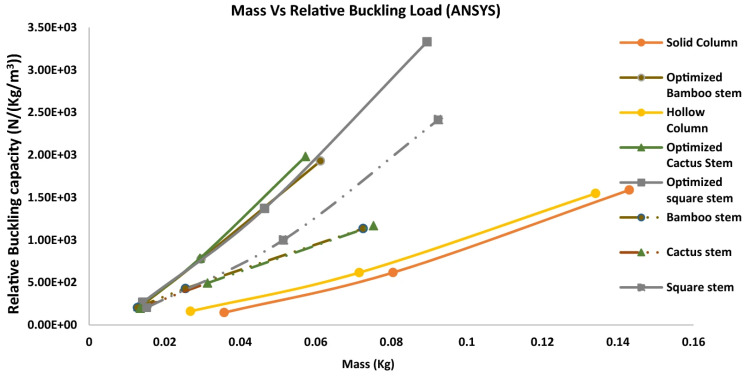
Mass of the specimens vs. relative buckling capacity of a solid column and optimized specimens in a hollow column (bamboo stem, cactus stem, and square stem). The optimized rods have almost double the buckling strength. Reproduced with permission from Guoqiang Li [97].

FEA was performed for the optimized rods shown in the graphic of Figure 11, results from which can be confirmed by the new rods exhibiting almost double the buckling strength compared to the first rods in the initial training dataset.

**Figure 12:**
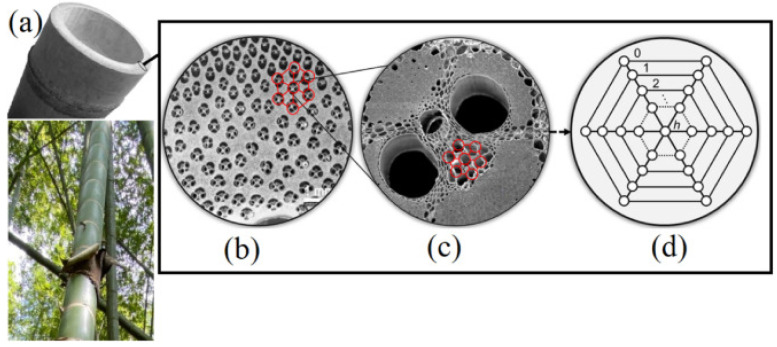
A cross-section of a bamboo plant (**a**), observed on SEM (**b**), and the comparison of the vascular system to the honeycomb structure (**c**,**d**). Reproduced with permission from Zaini Ahmad [88].

**Figure 13:**
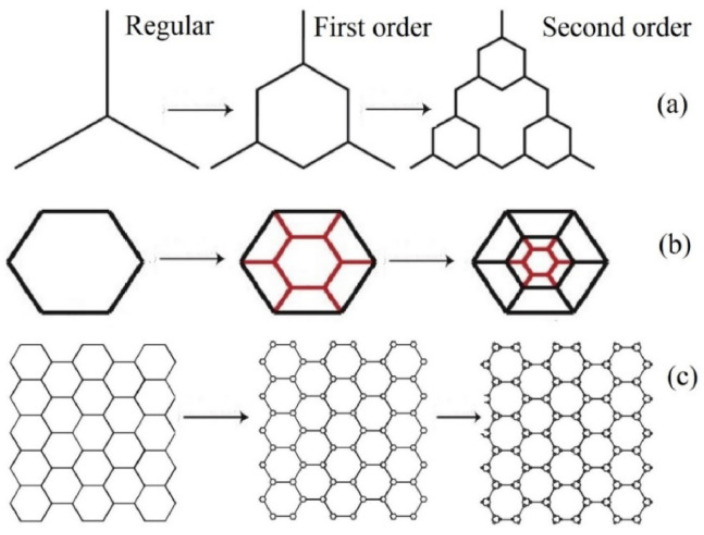
Possibility of applying fractals from regular structure, up to the first-order iteration and then to second order with a close-up to the intersection (**a**) of a single honeycomb (**b**) and on a honeycomb structure (**c**), as a possibility to increase mechanical strength on a specimen. Reproduced with permission from Zaini Ahmad [88].

In [88], referenced in Figure 12 and Figure 13, it is possible to see a comparison of honeycomb structures in the bamboo vascular system, which as been referenced as one of the best thin-walled structural designs.

In the same article, there is mention of fractal designs up to the second order and how honeycombs can be generated for specimens that underwent compression tests, where the value of SEA increased, as based on Figure 13.

## 9. Voronoi Diagrams

**Figure 14:**
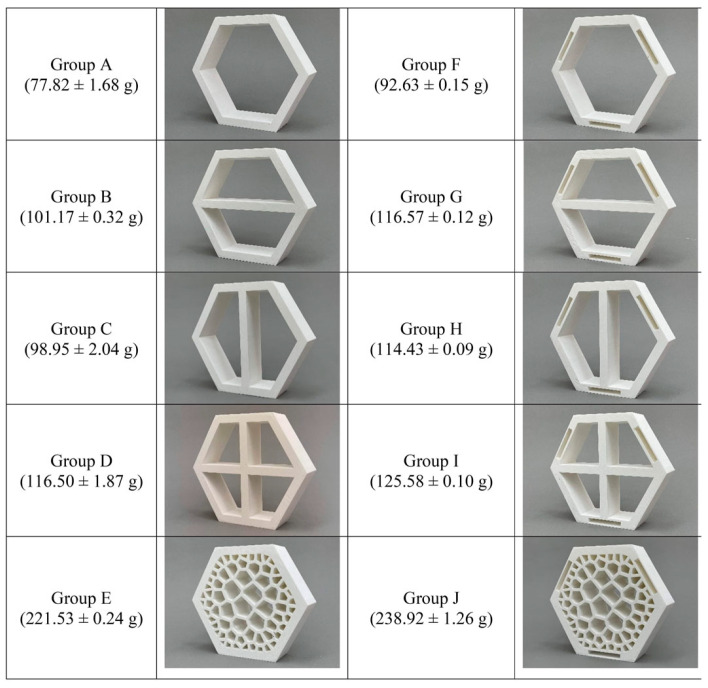
Average weight for each group of honeycombs; each group has different fillings. Reproduced from [91]. Used under Creative Commons CC BY license.

Another study filled 3D-printed combined honeycombs with porous fluid patterns, using Voronoi Diagrams to design the structures [91] that could support higher loads, as seen in Figure 14.

**Figure 15:**
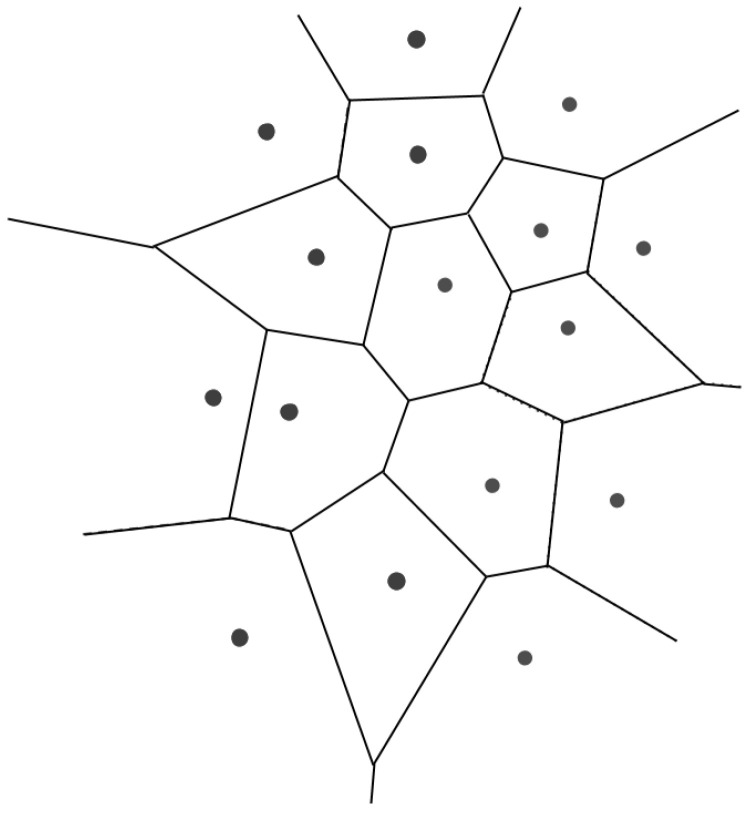
A Voronoi Diagram can be generated from random points [99], as similarly achieved in [100]. In this present figure, a Voronoi Diagram is represented. Reproduced from [99]. Licensed under the Creative Commons Attribution-Share Alike 4.0 International.

The Voronoi Diagrams, as exemplified in Figure 15, are computational geometries that allow many different organic studies from anthropologists on the influence of cultures, crystallographers on the structure of certain crystals, botanists on the development of the competition of plants, and even economists on the progression of the economy [98].

To generate Voronoi Diagrams, a Monte-Carlo simulation was used, just as was carried out in this paper [101], and then the authors used finite element analysis to determine the stress field. With this, they studied the regions with high stress that were further reinforced with seeds (to create Voronoi Diagrams).

Voronoi Diagrams were also used to reconstruct the cranial defects, with the aim of 3D printing SLM, using titanium for specific implants for each patient’s needs. The interest in implants based on natural formations, such as Voronoi structures, is focused on the development of porous implants that allow scaffolds for tissue growth. In [31], it is interesting to note that they used medically certified CAD modeling software (3-matic Medical v. 13.0) after designing an export to STL file. This study demonstrated the superiority of using asymmetrical and irregular porous models to mimic the human bone tissue. The intricate design of Voronoi structures demands the use of additive technologies, which would be harder with traditional methods. Here, the authors spot the challenge with the minimum thickness of 0.4 mm in SLM 3D printers. The melt pool is prone to defects, resulting in a sagging structure on the bottom regions of the printed model.

**Figure 16:**
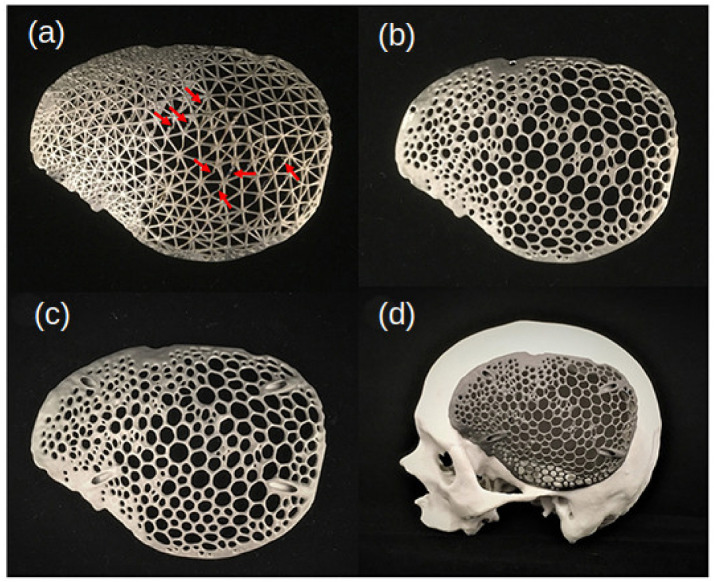
Three-dimensionally printed skull biomodel and the prostheses with selective laser melting (SLM) used in [31]: (**a**) wireframe pattern; (**b**) Voronoi pattern with flange screw fixation points; (**c**) Voronoi patterns with angular screw fixation points; (**d**) cranial prosthesis. Reproduced with permission from Thieringer Florian and Neha Sharma [31].

In Figure 16, (a) for the printed wireframe pattern, the red arrows are structural printing issues; (b) this structure is printed with the Voronoi pattern with flange screw fixation points; (c) this structure is also printed with Voronoi patterns but with angular screw fixation points; and (d) a cranial prosthesis customized for a specific skull was created [31].

**Figure 17:**
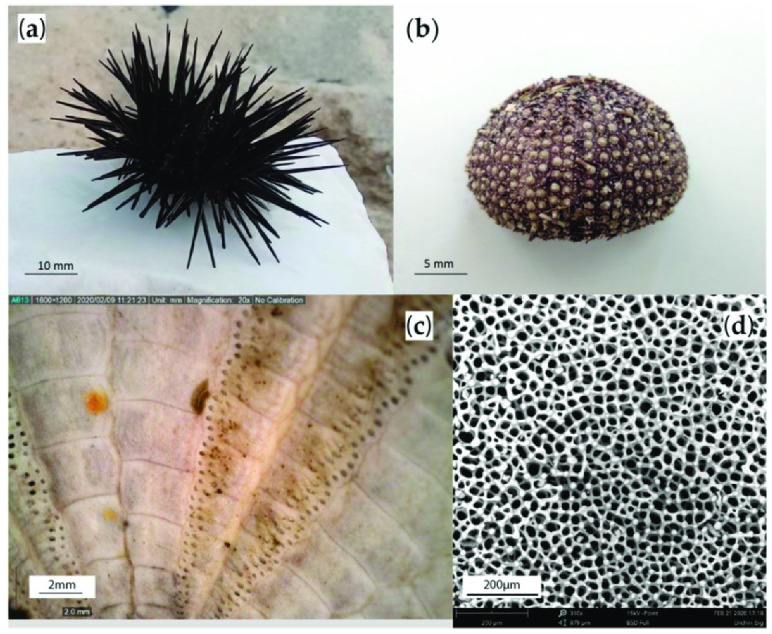
(**a**) Fresh sea urchin, (**b**) dried sea urchin, (**c**) the plates under an optical microscope, and (**d**) Voronoi microstructure observed under SEM microscope. Reproduced from [102], under the Creative Commons Attribution (CC BY 4.0) license.

There was also a study with Voronoi Diagrams that was inspired by Paracentrotus Lividus shells, also known as sea urchin (Figure 17). The authors observed the microstructure of this animal and applied it to 3D printing with PLA.

## 10. Microsctrutures and Biocomposites–Beetle Shell, Nacre, and Enamel

There are materials and nanocomposites of proteins and minerals that give superior strength such as bone, but nacre (mother pearl) and enamel (tooth surface) can also provide reinforcement. Seashells, bones, and teeth have laminated structures, enhancing mechanical properties [103,104,105,106,107,108].

Enamel is composed of long needle-looking types of crystals of 15–20 nm and is surrounded by a soft matrix. Bones have mineral crystal platelets embedded in a collagen matrix. The nacre has a pattern similar to breaks. This inspires researchers to not only produce this pattern on a macroscale, but also to produce composites, referring to embedded matrices. The ratio of mineral to matrix is 1:2. In [109], the importance of the nanoscale of these structures is discussed, and the researchers used a finite element analysis, showing that the stress field is more uniform as the thickness of the platelet decreases. They find this to be a drastic contrast to the classic macroscopic flaws. The most important conclusion of this study was that materials become insensitive to flaws when the structural size reaches its critical length. This is an important data point if there are goals to produce materials on a nanoscale. In [110], the minimum structural thickness of 250 µm is referred to as the lowest resolution, but the challenges of the laser powder bed (in spite of the laser being 60 µm) are not resolved. This theme is debated while discussing printing of these structures. It has been noted that the thermal cycling during printing can account for distortion and cause some failures. With this, challenges are accounted for during metal 3D printing while emulating these structures.

Helicoidal structures are another type found in nature as chitinous reinforcement in beetle, shrimp, and other crustaceans, showing some laminated angles, which, therefore, increases tolerance to damage. Some studies were made with a jetting technique of 3D printing, which demonstrated a relationship between the helical angle and mechanical properties. One conclusion is that the elastic modulus increases while increasing the laminate orientation angle [110].

The authors of [111] worked with microstructures with a laser additive 3D printer for compresssion tests of specimens. The effect of the densification behavior laser power and scanning speed on the relative density was studied. Velocity decreases as laser power increases, leading to the vapor depression instability. For example, more laser energy density intensifies the evaporation of Mg, contributing to a decrease in the densification degree. In this paper, lattice structures were produced on different laser powers, and the homogeneity of the molten pool size enhanced with the laser power was found to be between 375 W and 400 W, and it became worse when it reached 450 W. In terms of the mechanical properties, the study shows different lattices printed with different powers in the laser beam. The maximum load achieved was with 375 W, which is in the middle of other laser power ranges, as values of 350 W, 400 W, 425 W, and 450 W were also used. The printing speeds were all 3500 mm/s.

In the same paper, the different compressive properties of lightweight structures were compared based on previous investigations. The authors concluded that the bio-inspired lattice structure has an “excellent combination of lightweight and high strength” properties. They also noted that the studies conducted with SLM printers should take the power of the laser into account, as well as the temperature variable.

## 11. Organic Lattice Structures in Orthopedics

Medical implants have achieved many benefits with 3D printing. A patient will no longer be required to rely on off-the-shelf (OTS) solutions when custom-made implants based on their unique anatomy can be ordered, enabling better solutions in more complex and unique cases. The advantage of custom-made implants is the possibility to mimic a patient’s anatomy, improving the kinetic function. Custom-made implants might be more expensive, but they allow for faster surgeries, shorter recovery times, and a reduced risk of infections.

A practical example is restoring the chewing function to a patient, where additive manufacturing allowed for copying the anatomy and made it possible to have symmetry in the face, as well as printing part of the jaw in titanium and Ti-6Al-4V ELI powder, in a GE Additive Arcam EBM Q10plus system [112].

**Figure 18:**
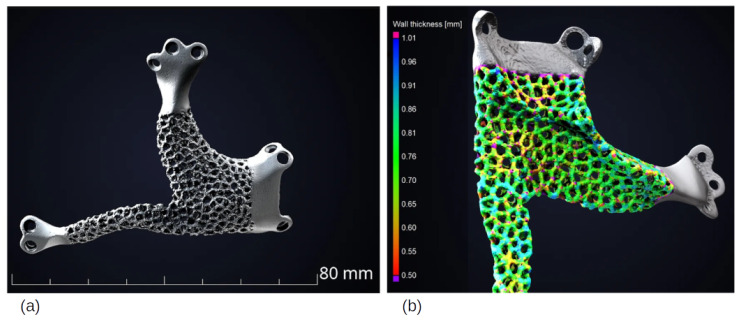
(**a**) Jaw implant in titanium, printed with Ti-6Al-4V ELI powder; (**b**) wall thickness analysis of porosity/inclusion. Adapted with permission from Sandra Engels [112].

In this case, as shown in Figure 18, lattices were used for the patient’s comfort as well to reduce the implant failure. Since titanium has a lower density, it reduces the weight discrepancy, with the weight of the implant approaching the real bone weight.

A wall thickness analysis was carried out to evaluate the connection between the regions, an essential step to preventing the implant from having broken struts and not exceeding tolerances by detecting cracks and inclusions, as well as critical pores [112].

Bones have porosity and the implants should have a similar structure, providing space to grow cells, tissues, blood vessels, and nerves. There have been discussions and controversies about the size of porosity as some papers present small sizes of porosity, like 100–400 μm, and others present between 50 and 125 μm. Smaller pores will induce the growth of cartilage and then new bone growth. Pore sizes greater than 350 μm allow the bone to grow directly. It has been noted that there is a need to use a resolution above 10 μm in body implants for complex components with fine structures [113].

Fine structures are the microscopic details with clinical or even functional relevance, which could affect the interaction with nearby tissues and biocompatibility. Authors of previous studies have indicated the need to reduce the resolution to 10 μm for these structures. Some works used pore sizes of 230–1000 μm, using a binder jetting 3D printer to create tricalcium phosphate scaffolds, printing in ceramics with a composition similar to bone. Studies were also conducted in metal, not only mimicking bone and its porosity but also its overall properties [110].

## 12. Biomimetics/Organic Shapes in Design, Architecture, and Automotive Industry

### 12.1. Biomimetics in Design

There are limitations to using traditional design, which requires milling machines and classical tools, whose form changes with angles, sharp edges, and flat surfaces due to the limitation of the tools. With AM, the array of possibilities changed, for example, with new designs in the artistic world [114]. This allows more freedom to obtain more complex and new designs with the help of 3D printing, and not basing biomimetics on functional purposes, but instead using AM technologies to obtain free forms, resembling organic shapes.

### 12.2. Biomimetics in Architecture

**Figure 19:**
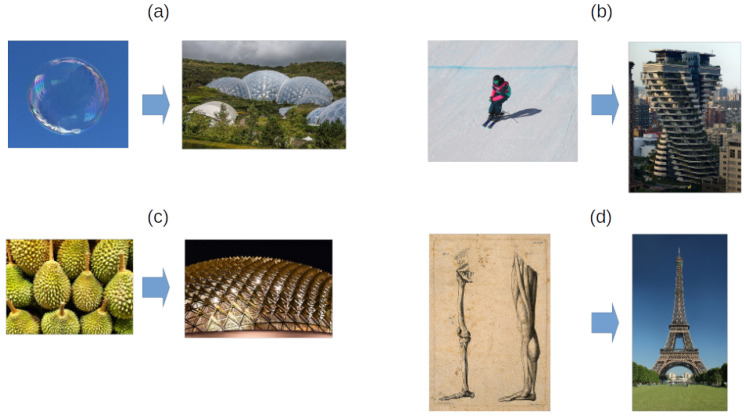
Biomimetic inspirations in architecture, (**a**) greenhouse inspired by a soap bubble; reproduced from PLBechly, 2017, licensed Creative Commons Attribution-Share Alike 4.0 International license [117], and reproduced from Ian Capper, 2017, licensed under the Creative Commons Attribution-Share Alike 2.0 Generic [118]. (**b**) Tao Zhu Yin Yuan (Agora Garden Tower) draws its design inspiration from the movements of a skier; reproduced from Yu tptw, 2023, licensed under the Creative Commons Attribution-Share Alike 4.0 International [119], and reproduced from Martin Rulsch, 2020, licensed under Creative Commons Attribution-Share Alike 4.0 International (CC BY-SA 4.0) [120]. (**c**) Singapore Arts Centre inspired by durian fruit; reproduced from Dudva, 2018, licensed under the Creative Commons Attribution-Share Alike 4.0 International [121], and reproduced from Dietmar Rabich/Wikimedia Commons/“Singapore (SG), Esplanade–Theatres on the Bay–2019–4693”/CC BY-SA 4.0 [122]. (**d**) Eiffel Tower inspired by human thigh bone; reproduced from A. Gajani after G. Guizzardi, 1814, Wellcome Library, London, licensed under the Creative Commons Attribution 4.0 International License (CC BY 4.0) [123], and reproduced from “Eiffel Tower, seen from the Champ de Mars, Paris, France”, Public Domain, by Benh LIEU SONG, [124]. All the contextual information was based on [125].

According to Figure 19, some architectural structures are based on nature [6,115,116]. There is a name for this nature-inspired architecture, and it is known as ’bioarchitecture’ [6]. Using some visual examples, as shown in Figure 19a, a bubble was the inspiration for an energy-efficient greenhouse using high-strength polymer known as ethylene tetrafluoroethylene (ETFE). In Figure 19b, Tao Zhu Yin Yuan (Agora Garden Tower) enhances seismic resilience by mimicking the way a skier shifts weight. In Figure 19c, based on the spiky layer of the durian fruit, this design protects the building from tropical heat. In Figure 19d, the human femur was the inspiration for the Eiffel Tower.

**Figure 20:**
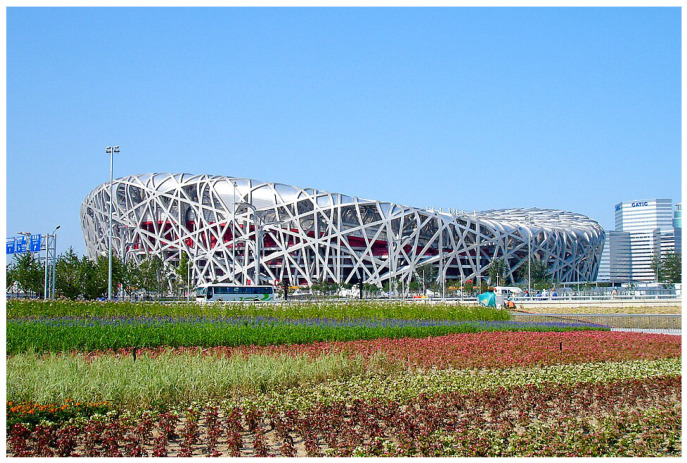
Beijing National Stadium, July 2008, inspired by a bird’s nest. Reproduced from Shujianyang, 2008, and licensed under the Creative Commons Attribution 4.0 International [127].

There is also a bio-inspired architectural structure: the Beijing National Stadium (Figure 20). Based on a bird’s nest, the steel facade and roof are detached from the concrete structure, which allows it to withstand seismic activity. It also provides harmony with nature [126].

**Figure 21:**
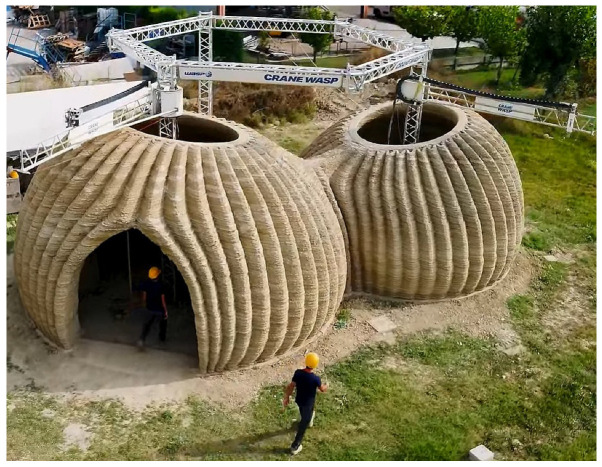
The Tecla houses that have been 3D-printed by two large synchronized robotic arms. Licensed under the Creative Commons Attribution 2.5 Generic license [130].

Contemporary architecture now includes models printed in concrete, enabling the realization of bold designs on a large scale, such as Tor Alva [128], the Tecla houses (Figure 21), and artificial reefs on the United Arab Emirates (UAE) coastline [129].

### 12.3. Organic Shapes with 3D Printing in Automotive Industry

Three-dimensional printing arrived in the automobile industry, and some expensive brands allow themselves to print parts as end-user products. This is the case of the 3D-printed brake calipers of the Bugatti Chiron, where they are stronger than usual calipers and lighter.This maximizes stiffness while reduces unsprung weight at the car’s corners [131]. The 3-million-dollar, 1500-horsepower car requires brakes capable to stop itself if driving at 420 km/h. Usually, these types of components are heavy, so Bugatti came up with with the idea to print it in titanium on a laser-sintered 3D printer. Because of titanium’s strength, it is impossible to use the same milling and forging techniques, having thicknesses between 1 mm and 4 mm, thanks to high-grade aerospace titanium alloy, which is an example of the necessity of 3D printing [132].

## 13. Discussion

### 13.1. General Discussion

Biomimetics in 3D printing analysis is an interest that has been studied in many fields such as biomedical implants and architectural mechanical properties; with this focus, many studies have been conducted. With the advance of technology to materialize objects like using 3D printing, which allows the ability to develop more complex shapes with different materials, different design strategies inspired by nature are made possible. Since using Voronoi, there has been a lot of inspiration from a variety of sources for engineering to increase the mechanical properties focusing on the optimization of materials, especially in terms of helicoid structures, from enamel to nacre, honeycombs, fractal progressions, xylem structures from plants, and bone porosity. With the analysis of keywords like biomimetic and 3D printing, as seen in Figure 2, this field is promising, and the interest in investigation has seen an exponential demand in the last years. As we discussed in this review and will further elaborate on, many studies can use LPBF 3D printing techniques. This technology can open the doors for many goals, like printing implants that require specific metallic organic tissue compatibility. It also allows, for example, for specific automotive parts to be obtained with metal printing technology, which cannot be achieved in another way. With this, nature-inspired structures can optimize the mechanical properties. Some interesting facts were observed when the 30° angles were prominent, as well as for the 3D printing inclination of the T-bone samples for tensile testings and the angle of the twisted specimens for compression [89]. But LPBF fabrication has some challenges; the supports need to be cut with saws, and other tougher techniques need to be used compared to FFF 3D printing. This means, for example, that thin walls need to be studied in order to determine how to print them and use supports. The pore sizes must be taken into consideration depending the goal of the printed item. The microscopic grain significantly influences the mechanical properties of the biomimetic structures. Further research is required for optimization of the processes for complex structures [75]. There is also the question of heating and thermal dissipation, one factor that was not accounted for in FFF 3D printers. As referred to in [133], thermal conduction while printing has a key role in the final printed item, and this variable and physical phenomena must be taken into account.

### 13.2. Further Research Opportunities

Studies have been conducted on the mechanical properties of 3D-printed organic shapes.

Several design strategies and experimental findings developed using Fused Filament Fabrication (FFF) techniques, such as those involving PLA, ABS, or SLA printing, can serve as a valuable foundation for further exploration in metal additive manufacturing. Specifically, there is a clear opportunity to replicate and adapt these geometric concepts and structural optimizations within the framework of laser powder bed fusion (LPBF) processes, potentially leveraging the mechanical and thermal advantages of metallic materials while building on prior knowledge from polymer-based studies.

Using the metallic powder, there is the chance to compare heat treatment specimens with no heat treatment. To fill the space of a structure, computational geometry using the Voronoi Diagrams can be employed, where the results can be very organic. The studies observed in this paper with Voronoi materials usually used PLA filament, and no studies with SLM technologies with metallic powder were conducted, which is an area that can be focused on in subsequent research. There is an opportunity to develop studies with dog-bone specimens using honeycombs or other nature-inspired structures in order to study strength in the LPBF 3D printer. Using composites is another desirable approach. For this review paper, not many studies combining biomimetics, composites, and LPBF printing were found, limiting the use of this 3D printing technique for ceramics, excluding biomimetic designs. LPBF is limited to only one material or some composite materials, presenting as a challenge, because for some biostructures worth studying for their mechanical properties, they have combined materials. Some authors expressed the need for developing multi-material LPBF technology, which would enhance the development of biomimetic structures with this technique [75]. As a part of new studies with LPBF, the specimens should undergo heat treatment and compare the results to specimens without heat treatment. The authors of [91] used honeycombs that are filled like porous bone, which can be studied with an LPBF 3D printer, and fractal progressions can be applied in honeycombs to study the increase in mechanical strength/material optimization. In [134], the authors studied the problem of hollowing solid objects, taking into consideration the optimization of the strength-to-weight ratio using Voronoi and finding an optimal interior tessellation to its maximal hollowing. Compression tests were conducted in FDM 3D-printed models. In this case, the inside volume of the models were determined with Voronoi, and they claimed that it was impossible for SLS, because, once the model is enclosed in this volume, the powder remains inside. In this case, simulations were not performed “due to the computational complexity it imposes”. In [135], the T-bone with Voronoi patterns was tested with 1mm/min, with the standard conditions [136,137]. Testing with LPBF technology should be conducted for comparison. The Table 6 presents a summary of several proposed studies to be carried out.

As shown in Table 7, the studies mentioned will be applied for LPBF technology, for tool steel, copper, or titanium, and for each one, it will be compared with heat treatment. There is also the possibility to study composites. The organic shapes can be designed based on other forms, moving away from the standard testing in the current state of the art [125]. There is an opportunity to study composites with LPBF 3D-printed specimens, with reference to other works that were not focused on biomimetics, for example, using the LSI technique [140].

Furthermore, the method using the LSI technique as complementary to LPBF was used with the combination of vacuum–pressure infiltration [141], where the infiltration temperature was 750 °C with an infiltration pressure at 8 MPa.

We can perform the experiments with the heat treatment on the 3D-printed specimens, conduct fatigue and Vickers tests with maraging steel (Material 1.2709), and compare the results of the biomimetic shapes to non-biomimetic shapes [77,142].

Other factors might influence the tests, like gas being heated by the laser and expanding outward and expelling the powder as well. Spatter particles influence the printing quality, because large particles cannot be melted completely. The results from [143] might help to train the eye and identify some defects and adjust the parameters of the printing.

The integration of biomimetics with additive manufacturing presents several technical and practical challenges that must be addressed to fully exploit its potential. Table 7 summarizes the key problems identified in recent studies, particularly those related to metal 3D printing techniques such as laser powder bed fusion (LPBF). These include limitations in mechanical testing, material constraints, process-induced defects, resolution barriers, and a lack of standardized evaluation methods. Understanding and overcoming these limitations is essential for advancing the field toward more reliable and biologically relevant applications in medicine, structural design, and industrial manufacturing.

**Table 7:** Key challenges in metal powder bed fusion (PBF) for biomimetics and related studies.

Challenge	Description	References
Multi-material/multi-function printing	Difficulty with printing multi-material structures like nacre-inspired composites exist, as well as scale and alignment challenges.	[144]
Residual stress	Rapid thermal gradients during printing induce significant internal stresses, potentially leading to part distortion and cracking.	[145]
Geometric fidelity	Complex natural geometries are hard to replicate with high accuracy due to defects, thermal distortion, and resolution limits.	[146,147]
Vascularization and internal channel replication	Reproducing internal biomimetic channels (e.g., for flow or implants) is challenging in metal AM.	[148]
Limits of printing volume	Maximum volume is usually in centimeters	[144]
Lack of fusion (LoF)	Inadequate or lack of energy input and excessive scan speed cause incomplete melting and weak bonding between layers or tracks.	[149]
Keyhole porosity/uncontrolled porosity	Excessive laser energy creates unstable vapor cavities (keyholes) that collapse and trap pores during solidification. Random porosity (LoF, gas) weakens parts; this contrasts with functional porosity in nature (like bone).	[150,151,152]
Gas-induced porosity	Entrapped gas within the melt pool or powder feedstock results in small, spherical pores affecting tensile and fatigue resistance.	[153,154,155,156,157]
Microstructural anisotropy	Directional solidification leads to columnar grain growth, resulting in anisotropic mechanical behavior.	[158]

Some challenges in metal powder bed fusion (PBF) can be observed in Table 7, for the biomimetics field. Keyhole formations in laser-based additive manufacturing have been extensively studied, with multiple predictive models proposed. However, a universally reliable and computationally efficient method to accurately predict their onset under varying process conditions remains elusive [159]. Techniques such as selective laser melting (SLM) and Electron Beam Melting (EBM), which fall under powder bed fusion, are characterized by rapid heating and cooling cycles that induce residual stresses. These stresses frequently result in geometric distortions like warping or dimensional shrinkage. While high accuracy is vital for medical components due to patient-specific requirements, industrial applications typically emphasize reliability and structural integrity over precision [160]. Based on prior practical experience, temperature gradients during the build process play a significant role in inducing distortions in the final geometry. Metallic materials are essential in this context, as they enable efficient thermal conduction of the laser energy, helping maintain dimensional accuracy. In certain cases, complex geometries must be printed in a horizontal orientation to avoid supports or allow thermal conductivity. When printed vertically, such parts require extensive support structures, which are particularly difficult to remove in small or intricate features. This not only increases post-processing effort and cost but may also negate the advantages of additive manufacturing, effectively resembling conventional subtractive fabrication. Furthermore, uncontrolled porosity and poor surface finish represent additional challenges, especially for components with fine details or inaccessible internal features. The irregular surfaces resulting from such limitations are difficult to polish or sand, further complicating the production of geometrically complex, high-precision parts. Elevated porosity levels were detected above the channels, likely resulting from irregular layer deposition associated with unsupported regions. The martensitic phase exhibits an almost random crystallographic texture, which may stem from the interplay between the applied energy density and the laser scanning strategy employed. Across all examined regions, the microstructure consists of cellular and columnar martensitic grains, displaying varying levels of refinement for the columnar grains of the martensitic phase [148]. The current size of printed samples is generally limited to the centimeter scale. Fabricating large-scale structures suitable for practical applications in fields such as automotive, armor, and aerospace engineering remains a significant challenge [144].

## Figures and Tables

**Table 1:** Biomimetic examples from pre-Industrial Revolution. Adapted from [1].

Year	Biomimetic Example
6000 BCE	Rock-Based Architecture
3000 BCE	Silk
2470 BCE	Pyramids
3 CE	Umbrellas
1500s	Study of Birds’ Flight
Mid. 1900s	The Crystal Palace

**Table 2:** Biomimetic examples from post-Industrial Revolution. Adapted from [1].

Year	Biomimetic Example
1903	Development of Air Planes
1955	Velcro
1966	Circular Economy
1986	Riblets
2010	Wind Turbines
2012	Gecko Feet
2014	Sustainable City Project
2022	Nanoscale Surface Texture

**Table 4:** Comparison of selected properties of biomaterials and their key applications. Data adapted from [45].

Metal	Strength–Weight Ratio (MPa/g/cm^3^)	Elastic Modulus (GPa)	Main Applications
Titanium (Ti)	∼100–120	∼110	Medical devices for bone support and oral reconstruction
Magnesium (Mg)	∼130–150	∼45	Implants designed to degrade naturally after healing, temporary action
Gold (Au)	∼5–10	∼80	Electronic systems for nerve interfacing and hearing enhancement
Stainless steel (316L)	∼25–30	∼200	Supportive devices for bone stabilization during healing, intended for removal
Platinum (Pt)	∼10–20	∼170	Electrodes, specialized implants
Cobalt–chromium (Co–Cr)	∼70–90	∼200–240	Load-bearing prostheses for major joint reconstruction

**Table 6:** Proposal of works in Trumpf 1000 3D printer for metallic biomimetic designs, and the papers for reference to techniques, studies, and state of the art as a basis for new approaches.

Study to Be Conducted	Equipment	References
S-N Curves with Honeycomb Bone-Shaped Specimens	Fatigue Testing Machine	[90]
Annealing Specimens	Heat Furnace	[81]
Tensile Test (Honeycomb Specimens)	Universal Testing Machine	[90,92]
Tensile Test (Porosity of Bone Structure/Voronoy)	Universal Testing Machine	[32,112]
Honeycomb Specimens Twisted in Z-Axis	Compression—Universal Testing Machine	[89]
Compression Testing (Fractal Honeycomb Specimens, Vascular Structures)	Universal Testing Machine	[30,88,91]
Hybrid Specimens	Universal Testing Machine, SEM, Durometer, Fatigue	[92]
Voronoi Tessellations	Universal Testing Machine	[31,135,138,139]
Micrographs with Tensile and Fatigue	Universal Testing Machine	[90]
Organic Shapes for Compression Testing	Universal Testing Machine	[125]

## Data Availability

The data are provided within the article.

## Nomenclature

3D	Three-Dimensional
ABS	Acrylonitrile Butadiene Styrene
AM	Additive Manufacturing
CAD	Computer-Aided Design
CoCrMo	Cobalt–Chromium–Molybdenum Alloy
DMLS	Direct Metal Laser Sintering
DLIP	Direct Laser Interference Patterning
ETFE	Ethylene Tetrafluoroethylene
FEA	Finite Element Analysis
FFF	Fused Filament Fabrication
LPBF	Laser Powder Bed Fusion
MF-3DP	Magnetic Field-Assisted 3D Printing
NIH 3T3	Fibroblast Cell Line Derived from Embryonic Mouse
OTS	Off The Shelf
PA	Polyamide
PETG	Polyethylene Terephthalate Glycol
PLA	Polylactic Acid
RMS	Root Mean Square
SEM	Scanning Electron Microscope
SLA	Stereolithography
SLM	Selective Laser Melting
SLS	Selective Laser Sintering
Ti6Al4V	Titanium Alloy with 6% Aluminum and 4% Vanadium
TPMS	Triply Periodic Minimal Surface
